# Cannabinoid receptor type 1 antagonist inhibits progression of obesity‐associated nonalcoholic steatohepatitis in a mouse model by remodulating immune system disturbances

**DOI:** 10.1002/iid3.338

**Published:** 2020-08-15

**Authors:** Chin‐Chang Chen, Zi‐Yu Chang, Fuu‐Jen Tsai, Shih‐Yin Chen

**Affiliations:** ^1^ Department of Traditional Chinese Medicine Chang Gung Memorial Hospital Keelung Taiwan, ROC; ^2^ Department of Anatomy, School of Medicine China Medical University Taichung Taiwan, ROC; ^3^ Institute of Traditional Medicine, School of Medicine National Yang‐Ming University Taipei Taiwan, ROC; ^4^ School of Chinese Medicine China Medical University Taichung Taiwan, ROC; ^5^ Department of Medical Research, Genetics Center China Medical University Hospital Taichung Taiwan, ROC; ^6^ Department of Medical Genetics China Medical University Hospital Taichung Taiwan, ROC

**Keywords:** cannabinoid receptor type 1 (CB1), immune system disturbances, mitogen‐activated protein kinase (MAPK)‐related inflammatory responses, nonalcoholic steatohepatitis (NASH)

## Abstract

**Scope:**

This study investigated whether AM251, a cannabinoid receptor type 1 (CB1) antagonist, ameliorates hepatic levels of metabolic abnormalities and inflammatory responses in a murine nonalcoholic steatohepatitis (NASH) model via reversal of disturbances in the immune system.

**Methods and Results:**

Fifteen‐week‐old male obese *db*/*db* mice were randomly assigned to the following two groups: no treatment and treatment with AM251 at 5 mg/kg for 15 days. C57BL/6J‐Lean mice were utilized as the control group. Plasma parameters, liver histopathology, and hepatic status were measured. For the in vitro study, macrophage‐derived RAW264.7 cells were cultured with AM251 or CB1 small interfering RNA (siRNA) before challenge with arachidonyl‐2′‐chloroethylamide (ACEA) or a high concentration of fatty acids (HFFAs). The *db*/*db* mice exhibited an increase in CB1 levels, lipid droplet accumulation, mitogen‐activated protein kinase‐related inflammatory responses, and macrophage and neutrophil infiltration in the liver tissues. Flow cytometry analysis revealed an elevation in macrophages and T helper cells, plus a decrease in natural killer T cells and regulatory T cells in the liver tissues of the *db*/*db* mice; treatment with 5 mg/kg AM251 reversed these changes. Moreover, in vitro experiments revealed that administration of 3.3 μM AM251 or CB1 siRNA prevented 1 mM HFFA‐ and 1 μΜ ACEA‐induced inflammatory cytokine protein expression in the RAW264.7 cells.

**Conclusion:**

These findings suggested that a blockade caused by CB1 reduced obesity‐associated NASH progression via correction of immune system dysregulations and elevated inflammatory responses in the liver tissues.

## Graphical abstract

These findings suggested a blockade caused by CB1 reduced obesity‐associated NASH progression via correction of immune system dysregulations and elevated inflammatory responses in the liver.

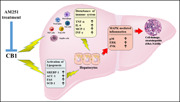

**Abbreviations**
ACEA: arachidonyl‐2′‐chloroethylamide
ALT: alanine transaminase
AST: aspartate transaminase
CB1: cannabinoid receptor type 1
ERK: extracellular signal‐regulated kinase
HFFA: high free fatty acid
HMNC: hepatic mononuclear cell
IFNγ: interferon γ
IL‐6: interleukin‐6
JNK: c‐Jun N‐terminal kinase
MCP‐1: monocyte chemotactic protein‐1
NAFLD: nonalcoholic fatty liver disease
NASH: nonalcoholic steatohepatitis
NK cell: natural killer cell
NKT cell: natural killer T cell
TG: triglycerides
TNF‐α: tumor necrosis factor‐α
Treg cell: regulatory T cell

## 1 INTRODUCTION

Nonalcoholic fatty liver disease (NAFLD) is a major public health concern worldwide. This illness encompasses a broad spectrum of liver disorders, ranging from simple steatosis to nonalcoholic steatohepatitis (NASH), which is characterized by hepatocellular lipid droplet accumulation, inflammation, and fibrosis.^1^ The increasing prevalence of NAFLD is associated with obesity, insulin resistance, and type 2 diabetes. In this regard, inflammation plays a crucial role in the pathogenesis of NASH, which results from the infiltration of inflammatory chemokines and cytokines secreted by adipose tissues, Kupffer cells, and lipid‐laden hepatocytes during obesity, such as tumor necrosis factor‐α (TNF‐α), monocyte chemotactic protein‐1 (MCP‐1), and interleukin‐6 (IL‐6).^2^ In addition, adiponectin is an adipocyte‐specific adipokine that regulates hepatic insulin sensitivity, and it is also anti‐inflammatory.^3^ Masarone et al^4^ have reported that adiponectin levels are downregulated in the serum of patients with NAFLD.

Disturbances in cytokines, adipokines, and the immune system can lead to NASH pathogenesis through crosstalk between the gut, adipose tissues, and the liver.^5^ A number of reports have determined that innate and adaptive immune cells participated in the pathogenesis of NASH.^6^, ^7^, ^8^, ^9^ It was reported that active hepatic Kupffer cells induce the release of TNF‐α to trigger NASH via monocyte recruitment.^6^ Rolla et al^7^ indicated that liver T helper 1 (Th1) cells were upregulated in a methionine‐choline‐deficient diet‐mediated NASH mouse model. Previous studies have revealed that the depletion of liver natural killer T (NKT) cells, which occurs in obese leptin‐deficient *ob*/*ob* mice.^8^ The regulatory T (Treg) cells are a subgroup of T cells that are either naturally occurring or inducible. It was reported that hepatic Treg cell numbers are reduced in the mouse models of NAFLD.^9^ Therefore, it may be plausible to modulate the disturbances to the immune system as a potential therapeutic strategy for NASH.

The endocannabinoid system, which is comprised of cannabinoid receptors, endogenous ligands, and the enzymes involved in endocannabinoid synthesis and degradation, affects metabolic regulation in the central nervous system and the peripheral organs.^10^ Among them, cannabinoid receptor type 1 (CB1) is predominant in the brain and is also present at lower levels in the peripheral tissues, such as adipose tissue, liver, and the skeletal muscle. It has been reported that a high‐fat diet (HFD) fed to mice led to an increase in hepatic levels of endocannabinoid anandamide, an endogenous ligand, and CB1 density, which further contributes to liver steatosis, dyslipidemia, insulin resistance, and leptin resistance.^11^ However, these phenomena are partially inverted by the genetic knockout or pharmacological blockade of the CB1.^11^ Irungbam et al^12^ also revealed that CB1 knockout treatment attenuated liver steatosis in hepatitis B surface protein (HBs)‐transgenic mice through repressing perilipin 2. Additionally, pharmacological blockade of CB1 inhibited hepatic elevated levels of oxidative/nitrosative stress and inflammation to further attenuated NAFLD.^13^ Furthermore, rimonabant, a CB1 antagonist, enhanced fatty acid β‐oxidation upon long‐term incubation in primary rat hepatocytes through activating phosphorylation of adenosine monophosphate‐dependent protein kinase (AMPK).* Our previous studies suggested an anti‐hepatic insulin resistance effect of the CB1 antagonist AM251; the mitochondrial function and gluconeogenesis improvements might have occurred in a forkhead box O1 (FoxO1)‐ and major urinary protein 1 (MUP1)‐dependent manner in both HFD‐induced obese mice and in a fatty acids‐laden hepatocyte model.^15^, ^16^ Furthermore, another CB1 antagonist, rimonabant, attenuated hepatic steatosis through repression of hepatomegaly, reduced hepatic TNF‐α levels, and increased plasma adiponectin levels in an obese Zucker *fa*/*fa* rat model.^17^ In addition, CB1 mediates cannabinoid effects in various types of T cells, dendritic cells, and macrophages, which express the highest basal levels of the Cnr1 gene.^18^ Genetic deficiency or pharmacological blockade of CB1 restrains lipopolysaccharides (LPS)‐induced fever in a mouse model through the reduction of proinflammatory cytokines released from macrophages and in the plasma.^19^ However, the role of CB1 in the dysregulation of hepatic immunity during NASH generation has not yet been elucidated. Therefore, we utilized an obese *db*/*db* mouse model to investigate whether AM251, a CB1 antagonist, ameliorates metabolic abnormalities through the re‐modulation of disturbances in the innate and adaptive immune system.

## 2 MATERIALS AND METHODS

### 2.1 RAW264.7 cell culture and preparation of HFFA and ACEA medium

RAW264.7 cell line, a mouse macrophage line, was purchased from the Food Industry Research and Development Institute (Hsinchu, Taiwan). RAW264.7 cells were cultured in Dulbecco's modified Eagle's medium (DMEM) (Gibco, Grand Island, NY). This medium contained 10% fetal bovine serum (FBS) (Gibco) and 1% Penicillin‐Streptomycin Solution (Invitrogen, Carlsbad, CA). Cultured cells were incubated at 37°C in a humidified environment with 5% CO_2_. Additionally, high free fatty acid (HFFA) was prepared with a high concentration of fatty acid and arachidonyl‐2′‐chloroethylamide (ACEA), a CB1 agonist (Sigma‐Aldrich, St Louis, MO) containing medium, as described in our previous study.^16^ Briefly, 1.0 mM HFFA medium was constituted at a 2:1 molar ratio of oleate‐palmitate mixture (two kinds of fatty acids purchased from Sigma‐Aldrich), and then added to fatty acid‐free BSA (Sigma‐Aldrich) (HFFA medium:BSA = 5:1 molar ratio). The ACEA medium was created utilizing 1.0 mM of ACEA stock solution, which was dissolved in DMSO and then added to the culture medium at a final concentration of 1.0 μM.

### 2.2 Animal study

Male C57BL/6J (Lean) and BKS.Cg‐Dock7m^+/+^ Lepr^*db*^/JNarl (*db*/*db*) mice were purchased from the National Laboratory Animal Center (Taipei, Taiwan), and housed under controlled temperatures at 22°C ± 2°C with a 12‐hour light/dark cycle and fed with a standard diet and water ad libitum. Fifteen‐week‐old mice were divided into three groups: (a) C57BL/6J‐Lean mice (*n* = 14), (b) *db*/*db* mice treated with the vehicle solution (7.7% DMSO, 4.6% Tween‐80, 87.7% saline) by once‐daily intraperitoneal (i.p.) injection (*n* = 14), (c) *db*/*db* mice treated with 5 mg/kg body weight of the CB1 antagonist, AM251 (dissolved with the vehicle solution, once‐daily i.p. injection) (Sigma‐Aldrich) for 15 days (*n* = 14). The mice were euthanized by CO_2_ inhalation before decapitation. Seven to eight mice of each condition were used to collect the plasma, epididymal white adipose tissues, and liver tissues, which measured tissue weight and stored at −80°C freezer for further analysis. Meanwhile, fresh liver tissue was taken in the other mice of each group for preparing hepatic mononuclear cells (HMNCs). All protocols were performed according to the Guide for the Care and Use of Laboratory Animals and approved by the Institutional Animal Care and Use Committee (IACUC) of China Medical University (IACUC permit no. 104‐34‐C‐1).

### 2.3 Plasma biochemical parameter assays

The plasma levels of triglycerides (TG), cholesterol, glucose, alanine transaminase (ALT), and aspartate transaminase (AST) were measured using commercially available diagnostic kits (all purchased from Randox Laboratories, Antrim, UK). In addition, the plasma levels of insulin were tested with an ELISA kit (Millipore Corporation, Billerica, MA), and adiponectin, TNF‐α, and IL‐6 levels were determined using commercial enzyme‐linked immunosorbent (ELISA) assays (purchased from R&D Systems, Minneapolis, MN). All plasma biochemical parameter assays were performed according to the manufacturer's instructions.

### 2.4 Histopathology and immunohistochemistry stain analysis

Liver tissues were fixed in 10% formalin, embedded in paraffin, and then cut into 5 μm thick sections. The sections were stained with hematoxylin and eosin (H&E) for histopathology analysis. Immunohistochemistry (IHC) staining was also performed; the slides were deparaffinized and rehydrated with decreasing percentages of ethanol, and sequentially incubated in 0.3% H_2_O_2_ with primary antibodies, including anti‐CD11b (dilution rate, 1:80; cat: ab133357; Abcam, Cambridge, UK), anti‐Neutrophil (dilution rate, 1:80; cat: ab53457; Abcam), and anti‐CB1 (dilution rate, 1:80; cat: ADI‐905‐708; Enzo Life Science Inc, PA). Subsequently, the biotinylated secondary antibodies and avidin‐biotin complex reagent were added, and color development was presented by 3,3′‐diaminobenzidine (DAB). All images were visualized and quantified using Panthera L Smart Light Microscope system (Motic, San Antonio, TX). In addition, the pathological lesion score of NAFLD was performed according to the histological scoring system for NAFLD.^20^

### 2.5 HMNC extraction and flow cytometry measurement

HMNCs were prepared according to Guebre‐Xabier's protocol using moderate modifications.^13^ In brief, HMNCs were isolated from the perfused liver tissues using collagenase (Sigma‐Aldrich) digestion along with gentle homogenization with a Teflon and glass tissue grinder for 2 minutes, followed by gradient centrifugation with Percoll (Sigma‐Aldrich) at 1500*g* for 15 minutes at room temperature for cell stratification. Subsequently, HMNCs were harvested in phosphate‐buffered saline (PBS) containing 1% FBS and then counted. Flow cytometry was performed using the following antibodies: rat anti‐mouse CD4 (cat. 553046), mouse anti‐mouse NK1.1 (cat. 561082) conjugated to fluorescein isothiocyanate (FITC), rat anti‐mouse F4/80 (cat. 565410) conjugated to PE, hamster anti‐mouse CD3 (cat. 553065) conjugated to PE‐CyTM5, and rat‐anti‐mouse CD25 (cat. 552880) conjugated to PE‐CyTM7 (all from BD eBiosciences, San Jose, CA). Immunoglobulins with isotypes corresponding to the above antibodies were conjugated to the appropriate fluorochromes for use as negative controls. HMNCs were stained via incubation with the various antibodies in FACS staining buffer at 4°C for 30 minutes to detect the surface antigens. The cells were washed twice with staining buffer, fixed with 2% formalin in PBS, and then analyzed using a BD FACSCalibur instrument (BD eBiosciences). The HMNCs which were stained with the FITC‐, PE‐, and PE‐Cy‐labeled antibodies were detected by FL1 (530 nm), FL2 (585 nm), and FL3 (650 nm), respectively. For each sample, 10 000 events were collected. All data were analyzed by FlowJo software (Tree Star lnc, Ashland, OR) and determined by a two‐parameter density plot with forward and side scatter profile. The percentages of macrophages, T helper cells, NKT cells, and Treg cells in HMNCs of each condition were determined by the gating sites.

### 2.6 Quantitative real‐time polymerase chain reaction

Total RNA was isolated from liver tissues using TRIzol reagent (Thermo Fisher Scientific, Waltham, MA) and the guanidinium thiocyanate‐phenol‐chloroform extraction method. Subsequently, synthesis of the complementary DNA (cDNA) was performed using a RevertAid First Strand cDNA Synthesis kit (Thermo Fisher Scientific). Quantitative real‐time polymerase chain reaction was conducted using the SYBR system with a LightCycler 1.5 apparatus (Roche Applied Science, Mannheim, Germany). The polymerase chain reaction reaction was carried out under the following conditions: 95°C for 10 minutes and then 45 cycles of 95°C for 15 seconds, 57°C for 30 seconds, and 72°C for 30 seconds. All data were normalized to glyceraldehyde‐3‐phosphate dehydrogenase (GAPDH). The primer sequences are presented in Table 1.

**Table 1:** Primer sequences used for quantitative real‐time polymerase chain reaction analysis

Gene	Forward	Reverse
CB1	CTACTGGTGCTGTGTGTCATC	GCTGTCTTTACGGTGGAATAC
SREBP‐1	ACTGTCTTGGTTGTTGATGAGCTGGAGCAT	ATCGGCGGAAGCTGTCGGGGTAGCGTC
ACC‐1	GGGACTTCATGAATTTGCTG	GTCATTACCATCTTCATTACCTCA
FAS	GCTGCGGAAACTTCAGGAAAT	AGAGACGTGTCACTCCTGGACTT
SCD‐1	CCGGAGAACCCCTTAGATCGA	TAGCCTGTAAAAGATTTCTGCAAACC
TNF‐α	TTGACCTCAGCGCTGAGTTG	CCTGTAGCCCACGTCGTAGC
IL‐6	GTACTCCAGAAGACCAGAGG	TGCTGGTGACAACCACGGCC
MCP‐1	AGCACCAGCACCAGCCAACTC	TGGATGCTCCAGCCGGCAACT
IFNγ	AGGCTCACGTCACCAAGTCCC	TGGTCTCGAAAGCTACGTGGGAGG
GAPDH	TCACCACCATGGAGAAGGC	GCTAAGCAGTTGGTGGTGCA

Abbreviations: ACC‐1, acetyl‐CoA carboxylase‐1; CB1, cannabinoid receptor type 1; FAS, fatty acid synthase; GAPDH, glyceraldehyde‐3‐phosphate dehydrogenase; IFNγ, interferon γ; IL‐6, interleukin‐6; MCP‐1, monocyte chemoattractant protein‐1; SCD‐1, stearoyl‐CoA desaturase‐1; SREBP‐1, sterol regulatory element‐binding factor‐1; TNF‐α, tumor necrosis factor‐α.

### 2.7 Western blot measurement

Western blot analysis was performed following the previous protocols.^17^, ^18^ In brief, total protein lysates from liver tissues were extracted in 0.5 mL of CelLytic M Cell Lysis Reagent (Sigma‐Aldrich) with 1% phosphatase inhibitor plus protease inhibitor cocktail (Sigma‐Aldrich), and then centrifuged at 13 000*g* for 20 minutes at 4°C. Subsequently, the protein lysates were separated using sodium dodecyl sulfate‐polyacrylamide gel electrophoresis (SDS‐PAGE) and transferred onto polyvinylidene fluoride (PVDF) membranes, followed by incubation with primary antibodies against CB1 (dilution rate, 1:500; cat: ADI‐905‐708; Enzo Life Science Inc), p‐p38 (dilution rate, 1:1000; cat: sc‐166182), p38 (dilution rate, 1:1000; cat: sc‐136210), p‐ERK (dilution rate, 1:500; cat: sc‐7383), ERK (dilution rate, 1:1000; cat: sc‐514302), p‐JNK (dilution rate, 1:500; cat: sc‐6254), and JNK (dilution rate, 1:1000; cat: sc‐7345) (all from Santa Cruz Biotechnology, Dallas, TX). Finally, horseradish peroxidase‐conjugated secondary antibodies were used for electrochemiluminescence detection. The data were normalized using β‐actin as an internal control.

### 2.8 Oil‐Red O stain and liver TG assay

Liver sections were prepared by embedding them in optimal cutting temperature (OCT) solution on dry ice; they were then cut and stained with Oil‐Red O reagent (Sigma‐Aldrich) to visualize fat droplet accumulation. Lipid extraction from the liver tissues was performed according to our previous protocol.^21^ Briefly, the liver tissues were homogenized in a 10× volume (w/v) of 2:1 chloroform/methanol, followed by centrifugation at 5000*g* at room temperature for 15 minutes. The lipid layer was washed with a 0.2× volume of 0.9% saline and centrifuged again at 5000*g* for 5 minutes. The lipid phase was completely dried and then dissolved in 0.2 mL of isopropanol containing 10% Triton X‐100. The TG concentration in the lipid mixtures was measured using a commercially available kit (Randox Laboratories, Antrim, UK) following the manufacturer's instructions.

### 2.9 CB1 small interfering RNA transient transfection

The CB1 gene was silenced using the small interfering RNA (siRNA) method according to the manufacturer's instructions (GE Dharmacon, Lafayette, CO). A scramble control or CB1 siRNA was added to sterilized RNA‐free water to prepare a concentration of 100 μM. Subsequently, RAW264.7 cells were transfected using DharmaFECT 1 transfection reagent (GE Dharmacon) combined with 100 nM CB1 siRNA duplex in DMEM medium without antibiotics, and then cultured in a humidified incubator with 5% CO_2_ at 37°C for 48 hours. Afterward, the cells were treated with the HFFA or ACEA medium for 24 hours before harvesting for quantitative real‐time PCR and Western blot analysis.

### 2.10 Statistical analysis

Data are expressed as mean ± SEM. Each group consisted of seven to eight mice and were compared using Student's *t* test and one‐way analysis of variance (ANOVA), followed by the Student Newman‐Keuls multiple‐range test. Differences for values of *P* < .05 were considered significant.

## 3 RESULTS

### 3.1 Effects of AM251 on body weight, epididymal white adipose tissues, liver weight, and plasma levels of biochemical and inflammatory cytokines in *db*/*db* mice

As indicated in Table 2 and Supplementary Data 1, the *db*/*db* mice initially presented with a significant increase in body weight, epididymal white adipose tissues, liver weight, food intake, and plasma levels of TG, cholesterol, glucose, insulin, ALT, AST, TNF‐α, and IL‐6, with decreased plasma adiponectin compared with the Lean mice. In addition, treatment with 5 mg/kg AM251 significantly reduced the body weight, epididymal white adipose tissues, and liver weight and reversed the phenomena of elevated TG, cholesterol, insulin, ALT, TNF‐α, and IL‐6 levels. Moreover, AM251 enhanced the reduced levels of adiponectin in the *db*/*db* mice, without an observed significant alteration in glucose and AST levels in comparison with the AM251 treated or untreated *db*/*db* mice.

**Table 2:** The impact of AM251 on body weight, liver weight, and plasma parameters of biochemical and inflammatory cytokines in *db*/*db* mice

	Lean	*db*/*db*	*db*/*db* + AM251, 5 mg/kg
Body weight, g	28.97 ± 3.89	59.19 ± 4.64*	50.43 ± 5.44**
Liver weight, g	4.58 ± 0.52	7.89 ± 0.33*	6.09 ± 0.75**
Epididymal white adipose tissue weight, g	1.07 ± 0.23	5.98 ± 0.75*	4.32 ± 0.86**
Plasma TG, mg/dL	87.93 ± 6.64	179.45 ± 10.68*	147.67 ± 17.45**
Plasma cholesterol, mg/dL	76.48 ± 15.64	130.55 ± 18.13*	100.68 ± 19.54**
Plasma glucose, mmol/L	2.53 ± 0.20	5.22 ± 0.43*	4.92 ± 0.52
Plasma insulin, mU/mL	14.53 ± 2.27	28.86 ± 3.53*	19.58 ± 5.84**
ALT, U/L	47.66 ± 7.38	235.48 ± 10.44*	157.63 ± 7.45**
AST, U/L	84.56 ± 6.19	155.47 ± 25.72*	138.86 ± 11.52
Plasma adiponectin, μg/mL	17.98 ± 2.78	8.73 ± 2.29*	13.58 ± 3.22**
Plasma TNF‐α, pg/mL	212.76 ± 34.58	853.37 ± 57.37*	568.37 ± 45.78**
Plasma IL‐6, pg/mL	23.89 ± 3.97	100.35 ± 8.45*	58.49 ± 6.67**

*Note:* Data are expressed as the mean ± SEM. Each group consists of seven to eight mice and compared using Student's *t* test.

Abbreviations: ALT, alanine transaminase; AST, aspartate transaminase; IL‐6, interleukin‐6; TG, triglyceride; SEM, standard error of the mean; TNF‐α, tumor necrosis factor‐α.

* *P*  < .05 compared with the Lean mice.

** P < .05 compared with the ob/ob mice.

### 3.2 Effects of AM251 on the pathohistological changes, inflammatory cell infiltration, and CB1 expression in liver tissues from the *db*/*db* mice

**Figure 1:**
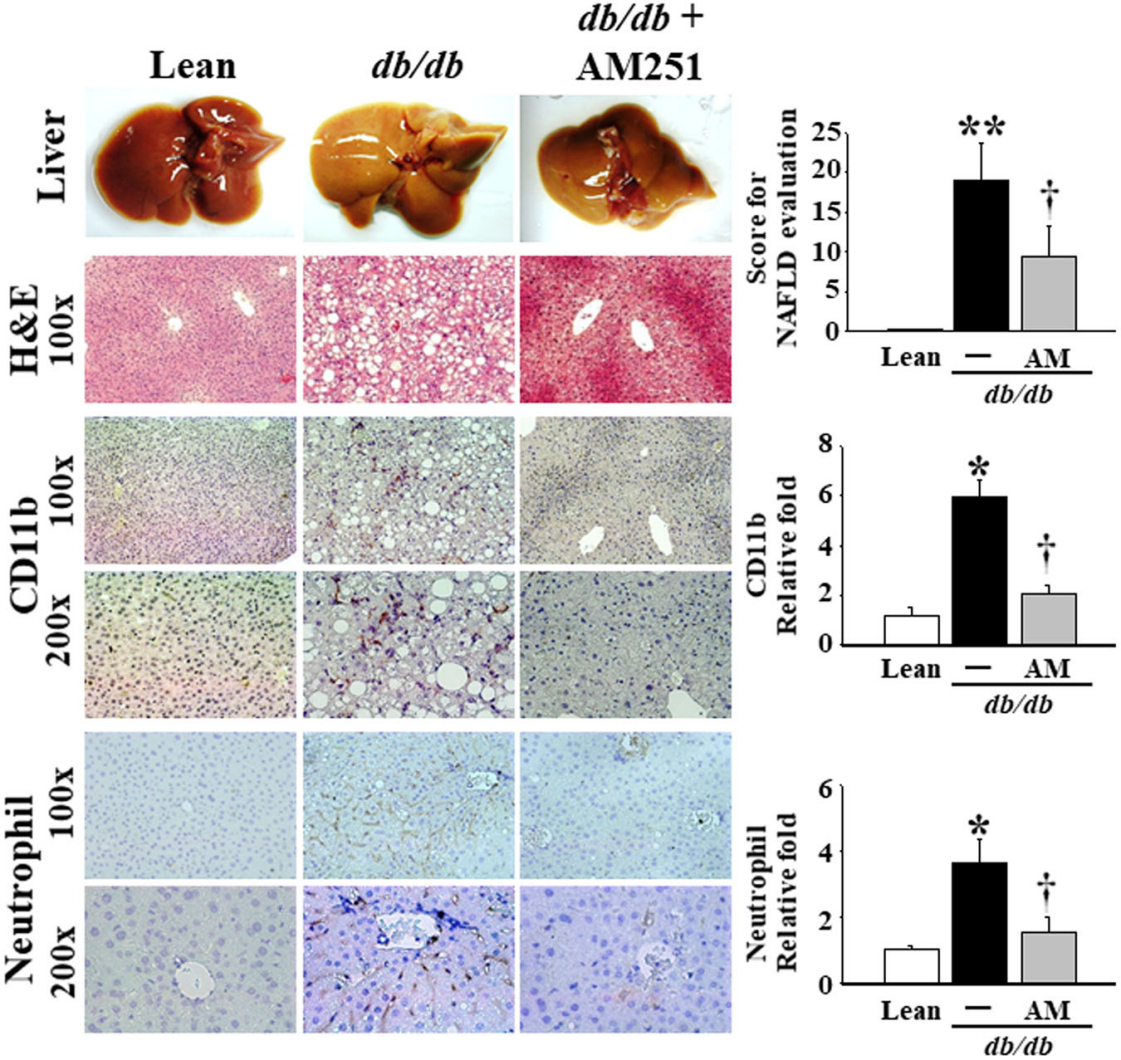
Effects of AM251 on tissue morphology and histopathological changes in the liver tissue of *db*/*db* mice. Liver tissues from the Lean control mice and *db*/*db* mice treated with or without 5 mg/kg AM251 were fixed in 10% formalin for H&E and IHC stain against CD11b (macrophages) and Neutrophils. Results for the H&E stain and IHC stain are shown at ×100 and ×200 magnification, and the dark brown color indicates the sites of CD11b and Neutrophils, respectively. The pathological lesion score of NAFLD was performed according to histological scoring system for NAFLD^20^ and the images of CD11b and Neutrophils IHC stains at ×200 magnification were quantified using Panthera L Smart Light Microscope system. *P < .05 vs Lean control mice (*n* = 8); ^†^ *P* < .05 vs *db*/*db* mice (*n* = 8). H&E, hematoxylin and eosin; IHC, immunohistochemistry; NAFLD, nonalcoholic fatty liver disease

**Figure 2:**
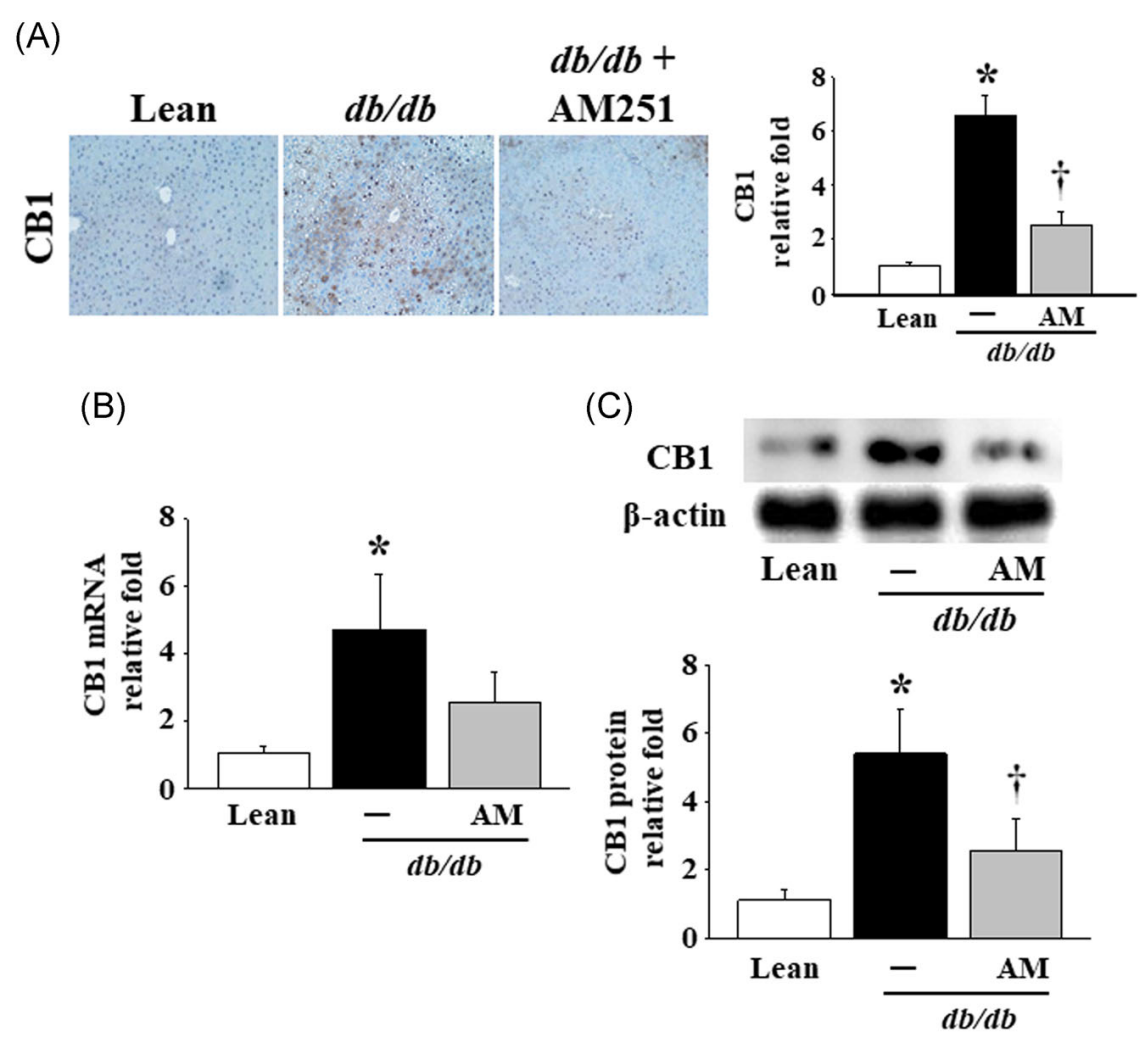
Effects of AM251 on the hepatic levels of CB1 in *db*/*db* mice. A, Representative result of the CB1 IHC stain in liver tissues of the Lean control mice and *db*/*db* mice treated with or without 5 mg/kg AM251. Dark brown color indicates the site of CB1, and the results are shown at ×200 magnification. The score of images was quantified using Panthera L Smart Light Microscope system. B, The mRNA and (C) protein levels in the liver tissues of mice were detected using quantitative real‐time polymerase chain reaction and Western blot analysis, respectively. *P < .05 vs Lean control mice (*n* = 8); ^†^ *P* < .05 vs *db*/*db* mice (*n* = 8). CB1, cannabinoid receptor type 1; IHC, immunohistochemistry; mRNA, messenger RNA

As shown in Figure 1, when compared with the Lean mice, the gross and pathohistological morphology of the liver tissues in the *db*/*db* mice appeared pale with no bright blood color, and severe degeneration was associated with micro‐ and macro‐vesicular fatty deposits (see H&E stain), accompanied by a massive infiltration of macrophages and neutrophils in the perivesicular lobule section (see CD11b and Neutrophil IHC stains). After the images of CD11b and Neutrophil IHC staining were quantified and the pathological lesion scores of NAFLD were performed in the liver tissues of mice, the results revealed that the significantly elevated levels of the pathological score occurred in the *db*/*db* mice in comparison of Lean mice. Meanwhile, these phenomena were obviously attenuated by treatment with 5 mg/kg AM251. In addition, the mRNA and protein levels of CB1 were significantly elevated in the liver tissues of the *db*/*db* mice (Figure 2); however, only the protein levels were significantly reduced by treatment with AM251 (Figures 2A and 2C).

### 3.3 AM251 administration suppressed lipid droplet accumulation and lipogenesis‐regulated factors in the liver tissues of the *db*/*db* mice

**Figure 3:**
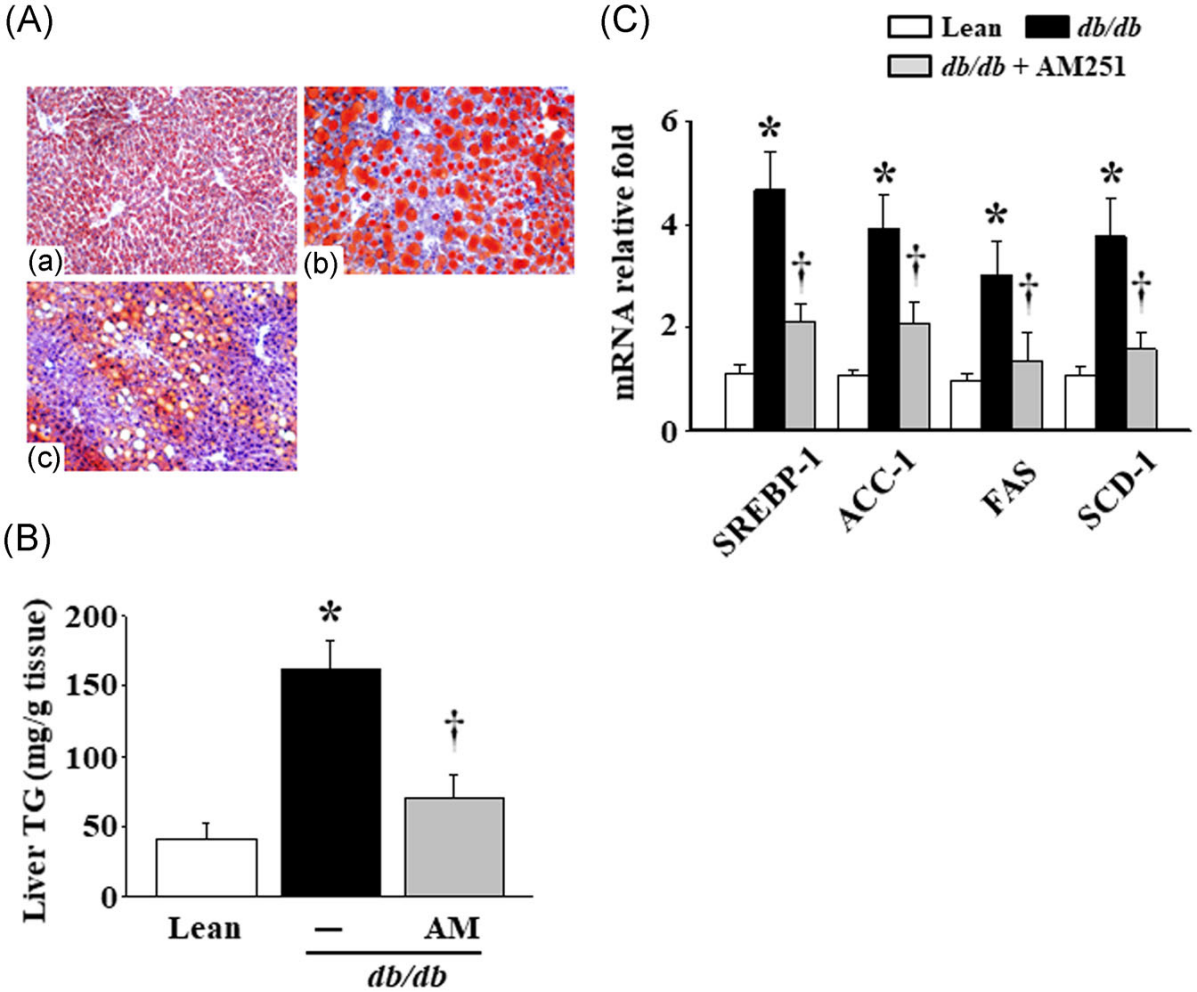
Effects of AM251 on lipid droplet accumulation and lipogenesis‐regulated factors in the liver tissues of the *db*/*db* mice. A, Liver tissues were embedded in an OCT medium and stained with Oil‐Red O reagent. The conditions were listed as (a) Lean control mice, (b) *db*/*db* mice, and (c) *db*/*db* mice treated with 5 mg/kg AM251. The results are shown at ×200 magnification. B, Liver TG levels were detected using colorimetric assay. C, The mRNA levels of lipogenesis‐regulated factors were measured using quantitative real‐time polymerase chain reaction. *P < .05 vs Lean control mice (*n* = 7); ^†^ *P* < .05 vs *db*/*db* mice (*n* = 8). ACC‐1, acetyl‐CoA carboxylase‐1; FAS, fatty acid synthase; mRNA, messenger RNA; OCT, optimal cutting temperature; SCD‐1, stearoyl‐CoA desaturase‐1; SREBP‐1, sterol regulatory element‐binding factor‐1; TG, triglycerides

The Oil‐Red O stain showed numerous red lipid droplets in the liver tissues of the *db*/*db* mice, and the number of lipid droplets decreased following the administration of AM251 (Figure 3A). This alteration was in parallel with the liver TG content (Figure 3B). Moreover, our results revealed that the mRNA levels of four lipogenesis‐regulated factors, sterol regulatory element‐binding protein‐1 (SREBP‐1), acetyl‐CoA carboxylase‐1 (ACC‐1), fatty acid synthase (FAS), and stearoyl‐CoA desaturase‐1 (SCD‐1), were significantly enhanced in the liver tissues of the *db*/*db* mice compared with those in the Lean mice. Following the 5 mg/kg AM251 administration, the previous increases in SREBP‐1, ACC‐1, FAS, and SCD‐1 mRNA levels were significantly curtailed in comparison with that of the *db*/*db* mice (Figure 3C).

### 3.4 AM251 treatment reduced the inflammatory response and the MAPK‐related factor expression in the HMNCs isolated from the liver tissues of the *db*/*db* mice

**Figure 4:**
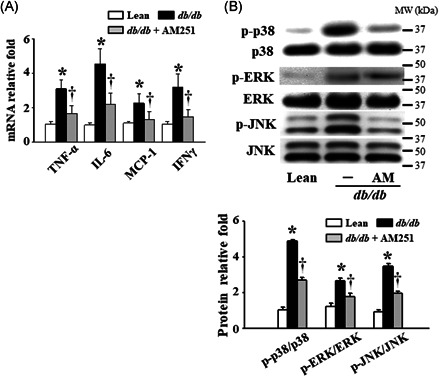
Effects of AM251 on the inflammatory response and MAPK‐related factor levels in the liver tissues of *db*/*db* mice. A, Hepatic mRNA levels of TNF‐α, IL‐6, MCP‐1, and IFNγ were measured by quantitative real‐time polymerase chain reaction. B, Hepatic protein phosphorylation levels of p38, ERK, and JNK were detected using Western blot analysis. *P < .05 vs Lean control mice (*n* = 7); ^†^ *P* < .05 vs *db*/*db* mice (*n* = 8). ERK, extracellular signal‐regulated kinase; IFNγ, interferon γ; IL‐6, interleukin‐6; JNK, c‐Jun N‐terminal kinase; MAPK, mitogen‐activated protein kinase; MCP‐1, monocyte chemoattractant protein‐1; mRNA, messenger RNA; MW, molecular weight; TNF‐α, tumor necrosis factor‐α

Arthur and Ley^22^ verified that mitogen‐activated protein kinase (MAPK)‐induced expression of nuclear factor κB (NF‐κB), TNF‐α, and interferon‐regulatory transcription factors could regulate the inflammatory response and innate immunity. Therefore, we detected the mRNA and protein levels of inflammatory response‐ and MAPK‐related factors from HMNCs. As expected, AM251 administration significantly decreased hepatic mRNA levels of TNF‐α, IL‐6, monocyte chemoattractant protein‐1 (MCP‐1), and interferon γ (IFNγ) in the *db*/*db* mice (Figure 4A). Our Western blot data showed that hepatic protein phosphorylation of p38, ERK, and JNK in the *db*/*db* mice was higher than that of the Lean mice. Meanwhile, AM251 treatment significantly reduced the protein phosphorylation of these factors in the liver tissues of the *db*/*db* mice (Figure 4B).

### 3.5 Effects of AM251 on macrophages, T helper cells, NKT cells, and Treg cells in the HMNCs isolated from the liver tissues of the *db*/*db* mice

**Figure 5:**
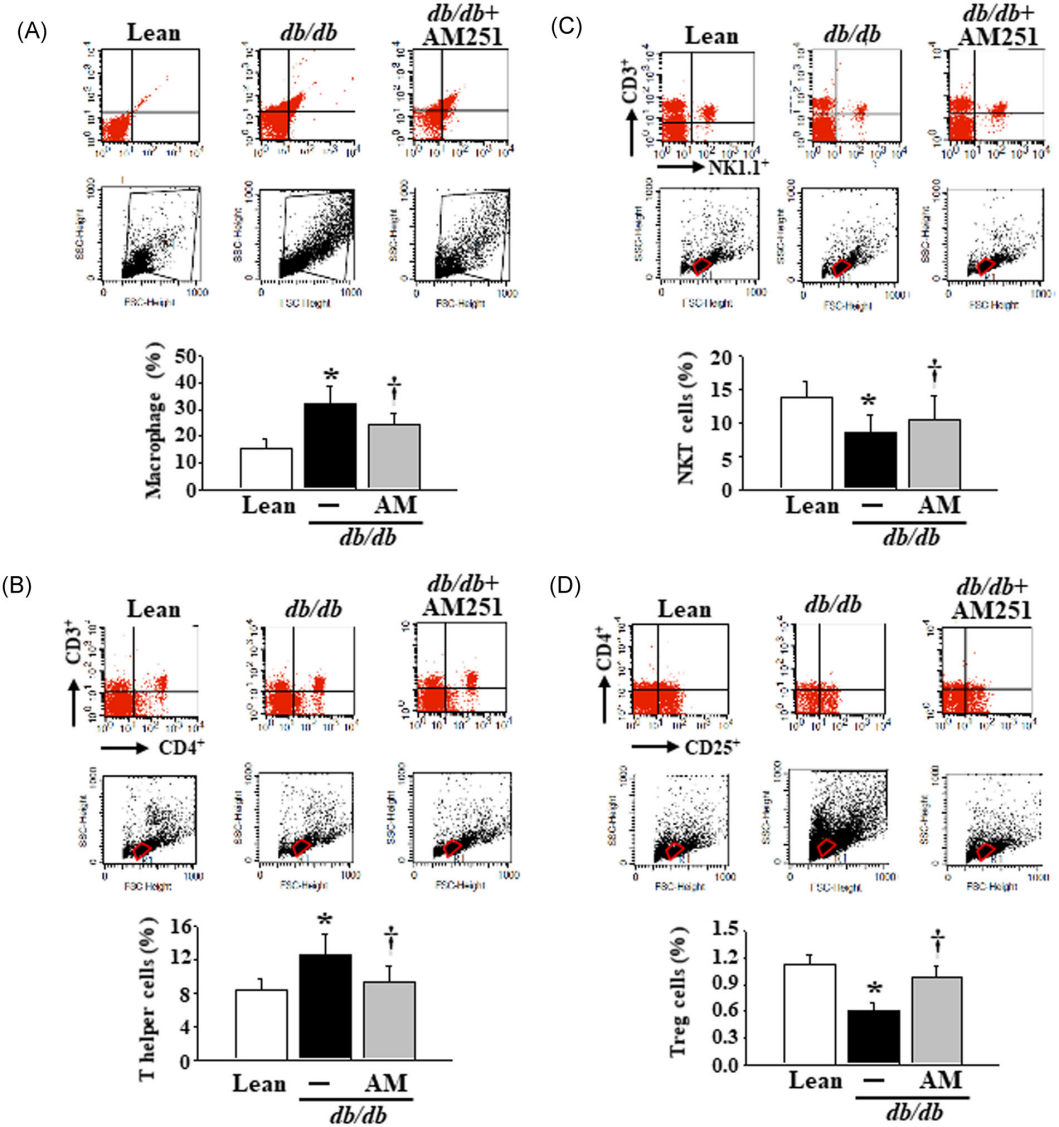
Flow cytometry analysis of the macrophages, T helper cells, NKT cells, and Treg cells in the Lean control mice and *db*/*db* mice treated with or without 5 mg/kg AM251. HMNCs were isolated from fresh liver tissues of mice before the execution of flow cytometry analysis. The axes on the flow cytometry dot‐plots were labeled at trapezoid shape with black color or round shape with red color. The expression of F4/80, CD3^+^CD4^+^, CD3^+^NK1.1^+^, and CD4^+^CD25^+^ was presented as a percentage of macrophages, T helper cells, NKT cells, and Treg cells, respectively. *P < .05 vs Lean control mice (*n* = 8); ^†^ *P* < .05 vs *db*/*db* mice (*n* = 8). HMNC, hepatic mononuclear cell; NKT, natural killer T; Treg, regulatory T

The flow cytometry data showed that the HMNCs from *db*/*db* mice revealed significantly elevated levels of macrophages (F4/80) (Lean control mice:*db*/*db* mice = 15.32 ± 15.47:32.87 ± 6.58 (%)) and T helper cells (CD3^+^CD4^+^) (Lean control mice:*db*/*db* mice = 8.38 ± 1.27:12.84 ± 2.36 (%)), as well as decreased levels of NKT cells (CD3^+^NK1.1^+^) (Lean control mice:*db*/*db* mice = 13.87 ± 2.36:8.47 ± 2.64 (%)) and Treg cells (CD4^+^CD25^+^) (Lean control mice:*db*/*db* mice = 1.12 ± 0.13:0.63 ± 0.08), in comparison with HMNCs in the Lean mice (Figure 5). Meanwhile, these effects were reversed by treatment with 5 mg/kg AM251 (Figure 5).

### 3.6 AM251 treatment or genetic silencing of CB1 inhibited HFFA‐ or ACEA‐induced CB1 protein expression and the inflammatory response in RAW264.7 cells

**Figure 6:**
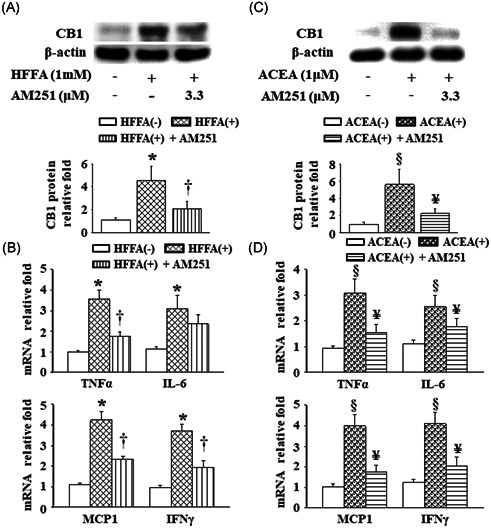
Effects of AM251 on HFFA or ACEA‐induced CB1 protein levels and the inflammatory response in RAW264.7 cells. A and C, The CB1 protein expression and (B and D) cellular mRNA levels of TNF‐α, IL‐6, MCP‐1, and IFNγ were analyzed using Western blot analysis and quantitative real‐time polymerase chain reaction, respectively. Raw264.7 cells were pretreated with 3.3 μM AM251 for 1 hour, followed by co‐treatment of 3.3 μM AM251 with 1 mM HFFA or 1 μM ACEA for 24 hours, separately. Data are presented as the mean ± SEM from three independent experiments. *P < .05 vs HFFA (−); ^†^ *P* < .05 vs HFFA (+); ^§^ *P* < .05 vs ACEA (−); ^¥^ *P* < .05 vs ACEA (+). ACEA, arachidonyl‐2′‐chloroethylamide; CB1, cannabinoid receptor type 1; HFFA, high free fatty acid; IFNγ, interferon γ; IL‐6, interleukin‐6; MCP‐1, monocyte chemoattractant protein‐1; mRNA, messenger RNA; SEM, standard error of the mean; TNF‐α, tumor necrosis factor‐α

**Figure 7:**
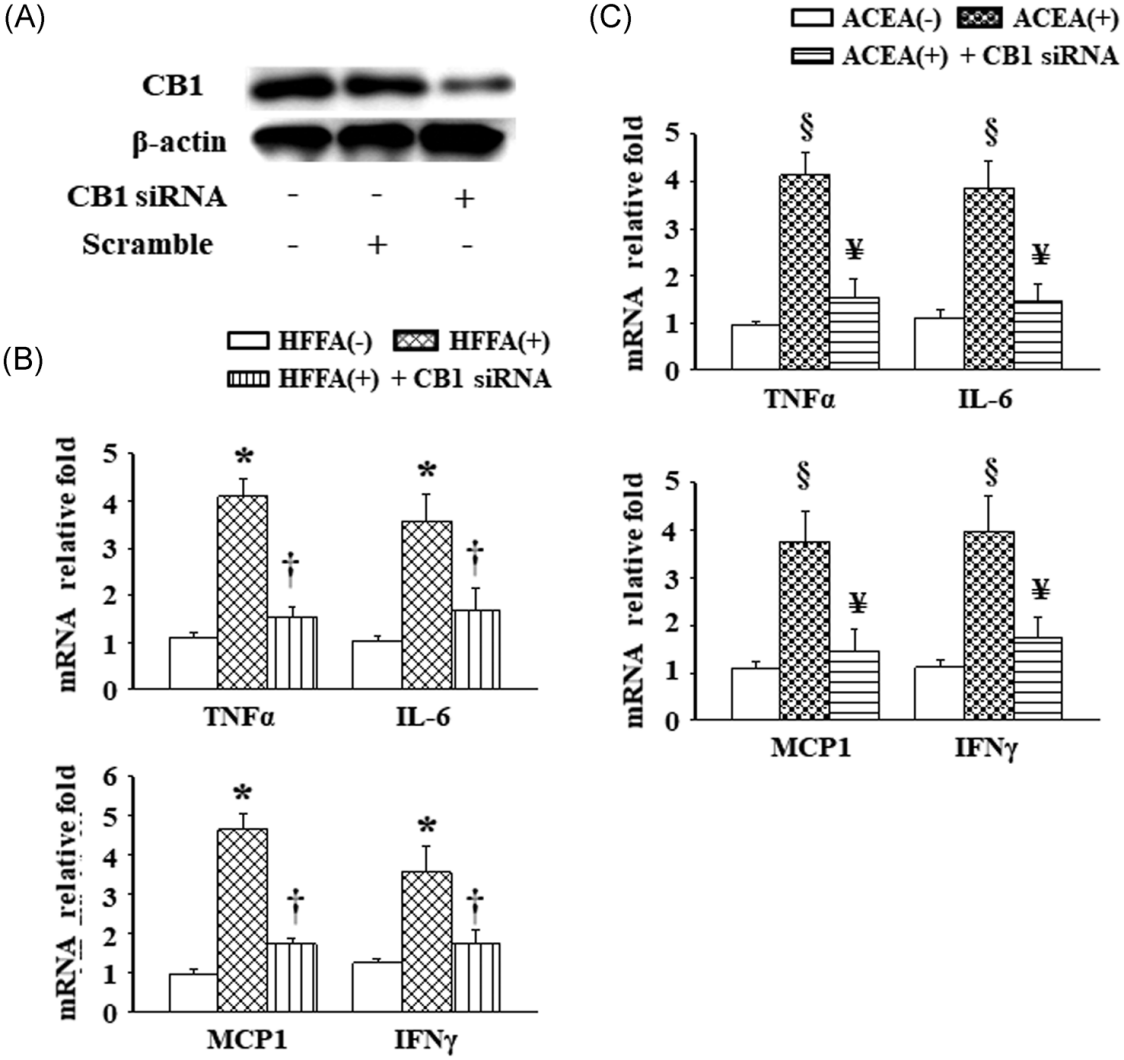
Effects of genetic silencing of CB1 on HFFA‐ or ACEA‐induced inflammatory responses in the RAW264.7 cells. A, Changes in expression of the CB1 protein after CB1 siRNA transfection in RAW264.7 cells. B and C, Cellular mRNA levels of TNF‐α, IL‐6, MCP‐1, and IFNγ were measured after CB1 gene silencing, followed by exposure to 1 mM HFFA or 1 μM ACEA for 24 hours. Values are presented as the mean ± SEM from three independent experiments. *P < .05 vs HFFA (−); ^†^ *P* < .05 vs HFFA (+); ^§^ *P* < .05 vs ACEA (−); ^¥^ *P* < .05 vs ACEA (+). ACEA, arachidonyl‐2′‐chloroethylamide; CB1, cannabinoid receptor type 1; HFFA, high free fatty acid; IFNγ, interferon γ; IL‐6, interleukin‐6; MCP‐1, monocyte chemoattractant protein‐1; mRNA, messenger RNA; siRNA, small interfering RNA; SEM, standard error of the mean; TNF‐α, tumor necrosis factor‐α

Liver Kupffer cells and/or recruited infiltrating macrophages to play a vital role in the progression of NASH.^23^ To mimic the elevated hepatic levels of TG or the CB1 protein in the *db*/*db* mice, RAW264.7‐derived macrophages were challenged in HFFA‐ or ACEA‐containing medium. As depicted in Figures 6A and 6C, the CB1 protein expression was markedly upregulated in the cells challenged with 1 mM HFFA or 1 μM ACEA in comparison with cells cultured in HFFA‐ and ACEA‐free medium. Furthermore, these effects were inhibited by treatment with 3.3 μM AM251. Moreover, similar results occurred with the cellular TNF‐α, IL‐6, MCP‐1, and IFNγ mRNA levels (Figures 6B and 6D). Subsequently, CB1 siRNA transfected RAW264.7 cells showed that the CB1 protein levels were reduced by about 70% after transfection (Figure 7A). Furthermore, pre‐incubation with CB1 siRNA significantly suppressed 1 mM HFFA and also 1 μM ACEA‐induced mRNA levels of TNF‐α, IL‐6, MCP‐1, and IFNγ (Figure 7B,C).

## 4 DISCUSSION

Lipotoxicity and glucotoxicity act as key elements in both the development of simple steatosis and the progression of NASH, because during these conditions, the hepatocytes are exposed to high concentrations of lipids and carbohydrates, respectively. It is noteworthy that the progression of NASH follows the “two‐hit hypothesis”; the first hit is the appearance of steatosis, followed by a second hit leading to inflammation, hepatocyte damage, and fibrogenesis.^24^ Therefore, immune and inflammatory pathways play key roles in the pathogenesis of NASH. The present study demonstrated that the pharmacological blockade of CB1 improved hyperlipidemia (increased plasma TG and cholesterol levels), hyperinsulinemia (increased plasma insulin level), and increased inflammatory cytokine‐mediated NASH development in the obese *db*/*db* mice. Thus, this blockade also reversed the elevated levels of macrophages and T helper cells, and decreased the NKT cells and Treg cells in the liver tissue, and might further restrain the MAPK signal phosphorylation and inflammatory responses, including TNF‐α, IL‐6, MCP‐1, and IFNγ. Furthermore, both HFFA and ACEA induced the inflammatory response in the macrophage‐derived RAW264.7 cells; however, this was suppressed by the administration of AM251 or the genetic silencing of CB1.

A previous study indicated that CB1 blockade, rimonabant, reduced obesity‐mediated hepatic steatosis due to suppression of hepatic proinflammatory cytokine levels in both diet‐induced obese mice and obese Zucker *fa*/*fa* rats.^17^ Our study revealed that the NASH development trend in the obese *db*/*db* mice was accompanied by an inflammatory response in the plasma and liver tissue coupled with disturbances in innate and adaptive immune populations in the liver tissue (see Figures 1, 4, and 5). The results were similar to those of previous studies.^6^, ^7^, ^8^, ^9^ Furthermore, it was reported that another blockade of CB1, AM281, suppressed bone marrow‐derived macrophage infiltration, and ameliorated inflammation and fibrosis in carbon tetrachloride‐induced murine liver injury.^25^ Our study is the first to report that AM251 treatment neutralized obesity‐induced hepatic levels of immune dysregulation and the MAPK‐associated inflammatory response, and further contributed to NASH progression in obese *db*/*db* mice.

Adiponectin is also involved in controlling immune responses.^26^ Hu et al^27^ predicted that the reduction of adiponectin acts as an indicator of necroinflammatory forms of NAFLD. Moreover, it inhibited macrophage inflammatory protein‐1β (MIP‐1β) and IL‐7, two proinflammatory cytokines, in tissue explants, isolated adipocytes, and stromal‐vascular cells in omental adipose tissue samples from obese human subjects.^28^ Similarly, our results revealed that decreased plasma adiponectin levels and elevated inflammatory cytokine expression levels in the plasma and liver tissue were reversed following the administration of AM251. We speculated that adiponectin might be a target candidate for CB1‐mediated dysregulation of hepatic immunity in the progression of NASH based on the results of our murine model. However, this concept requires further investigation and research. In addition, Leptin receptor‐deficient *db*/*db* mice served as a NAFLD/NASH model.^29^ Our *db*/*db* mouse model results revealed that the macrophage infiltration, increased inflammatory responses, and immune system dysregulation in the liver tissues, is in agreement with the experimental observations by Elinav et al,^30^ Ni et al,^31^ and Onodera et al.^32^ Therefore, it seems reasonable to conjecture that adiponectin exerted beneficial effects toward the reduction of inflammation and dysregulation during the immune response in the NASH development. Despite, a proper lean littermate (heterozygotes) of *db*/*db* mouse is *Lepr* ^*db*^/+ mouse. It is a pity that we cannot purchase the *Lepr* ^*db*^/+ mouse in Taiwan. However, we find that the genetic background of BKS.Cg‐Dock7m^+/+^ Lepr^*db*^/JNarl mouse is C57BLKS/J mouse, and C57BLKS/J is closely related to C57BL/6J (The Jackson Laboratory website, https://www.jax.org/strain/000642). Therefore, we used the male C57BL/6J mouse as a lean control of *db*/*db* mouse in this study.

Rajesh et al^33^ reported that CB1 played an important role in the pathogenesis of diabetic cardiomyopathy by facilitating p38/JNK MAPK activation, angiotensin II type 1 receptor signaling, and inflammation in mice. Macrophages and dendritic cells, two types of innate immune cells, were crucial mediators of the inflammatory response via pattern recognition receptor (PRR)‐linked activation of MAPKs and NF‐κB.^22^ Our current results showed that elevation of MAPK protein phosphorylation, including p38, ERK, and JNK, and inflammatory cytokine mRNA expression, including TNF‐α, IL‐6, MCP‐1, and IFNγ, in the liver tissues of *db*/*db* mice was restrained by AM251 administration (see Figure 3). Thus, we speculated that the increased hepatic Kupffer cells and/or recruited infiltrating macrophages implicated in MAPK cascade activation and inflammatory responses might function in a CB1‐dependent manner in the NASH model. However, our results had not evidenced a direct relation between MAPK signal phosphorylation and inflammatory responses in the hepatic immune cells of *db*/*db* mice. The second limitation is that 5 mg/kg AM251 administration could significantly suppress food intake (see Figure S1B) and decrease the weight of epididymal white adipose tissue (see Table 2) to further cause reduction of body weight in our *db*/*db* mice. It is well known that metabolic abnormality, including NAFLD may be improved by reducing body weight and visceral adipose tissue mass in obesity. Therefore, we could not prove the evidence that AM251 treatment directly reversed immune system disturbances to inhibit the progression of NASH forms our current results. In turn, decrease of disturbances in the innate and adaptive immune system may also be caused by improving metabolic abnormality after AM251 treatment in *db*/*db* mice. Furthermore, orphan G‐protein coupled receptor, GPR55, was an atypical cannabinoid receptor for numerous endogenous and synthetic cannabinoids.^34^ Kapur et al^35^ demonstrated that AM251 is not only a CB1 inverse agonist/antagonist but also a GPR55 agonist. Of note, it has been recently reported that liver GPR55 is increased in the liver of patients and mouse models with NAFLD, and further participating in the progression of NASH.^36^ It seems to be a contradiction between AM251 and the role of GPR55 in the therapy of NASH. However, our results could not provide related information now. To verify this hypothesis, RAW264.7 macrophage‐derived cells were used as an in vitro model with HFFA or ACEA challenge to stimulate the inflammatory response; these phenomena were neutralized by treatment with AM251 or genetic silencing of CB1. Of note, many CB1 antagonists/inverse agonists suppressed food‐motivated behaviors, but neuropsychiatric side‐effects such as anxiety, depression, and suicidal ideation were also reported.^37^ The limitation of AM251 using is that it also induced the anxiogenic effects in the elevated plus maze test, an anxiety‐related measurement, in the rodents^38^, ^39^ may be through inducing c‐Fos immunoreactivity in the central amygdala, dorsal striatum, and nucleus accumbens shell region.^39^

**Figure 8:**
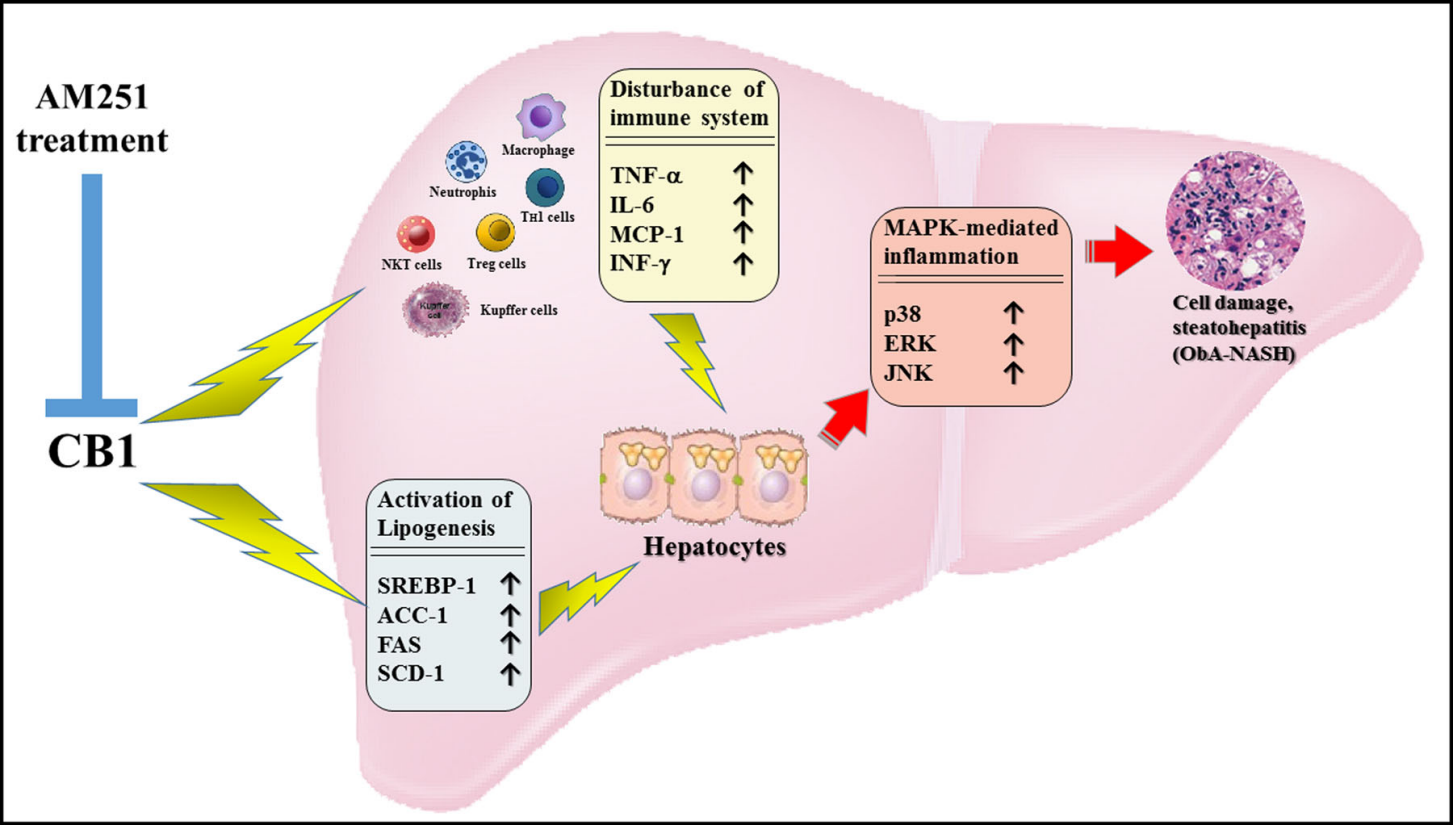
Schematic representative hypothesis of CB1 antagonist AM251 treatment to decrease dysregulation of the immune system and elevation of MAPK‐mediated inflammatory responses in our ObA‐NASH model. CB1, cannabinoid receptor type 1; MAPK, mitogen‐activated protein kinase; ObA‐NASH, obesity‐associated nonalcoholic steatohepatitis

In conclusion, a better understanding of the mechanisms of immune dysregulation may help reverse inflammatory infiltrate hepatitis. All of our findings suggested that CB1 antagonist treatment reduced obesity‐associated NASH progression via reversion of immune system dysregulation and elevation of MAPK signal phosphorylation and the inflammatory responses in the liver tissue. As such, a schematic hypothesis of the AMM251‐induced anti‐NASH effect is shown in Figure 8. Nevertheless, the underlying molecular mechanisms involved and the regulatory network require further investigations.

## CONFLICT OF INTERESTS

The authors declare that there are no conflict of interests.

## AUTHOR CONTRIBUTIONS

SYC and CCC conceived and designed the study. SYC, CCC, ZYC, and FJT analyzed and interpreted the data. SYC and CCC participated in the drafting of the manuscript. SYC, CCC, ZYC, and FJT critically revised the manuscript for important intellectual content. All authors read and approved the final manuscript.

## Supporting information

Supporting information

Supporting information
