# Supplementary figures and images for: Cannabinoid receptor type 1 antagonist inhibits progression of obesity‐associated nonalcoholic steatohepatitis in a mouse model by remodulating immune system disturbances
Source: Immun Inflamm Dis. 2020 Aug 15;8(4):544–58. doi: 10.1002/iid3.338 (PMC7654409; doi:10.1002/iid3.338)

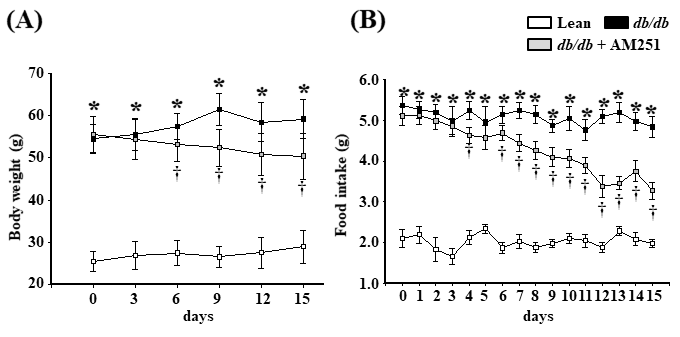

Supplement: Supplementary file 1 — Supporting information [file IID3-8-544-s001.tiff]
