# Supplementary material for: Cannabinoid receptor type 1 antagonist inhibits progression of obesity‐associated nonalcoholic steatohepatitis in a mouse model by remodulating immune system disturbances
Source: Immun Inflamm Dis. 2020 Aug 15;8(4):544–58. doi: 10.1002/iid3.338 (PMC7654409; doi:10.1002/iid3.338)
Supplement: Supplementary file 2 — Supporting information [file IID3-8-544-s002.docx]

1] Brunt, E. M., Kleiner, D. E., Wilson, L. A., Belt, P. et al., Nonalcoholic fatty liver disease (NAFLD) activity score and the histopathologic diagnosis in NAFLD: distinct clinicopathologic meanings. *Hepatology* 2001, 53, 810-820.

[2] Marra, F., Gastaldelli, A., Baroni, G. S., Tell, G. et al., Molecular basis and mechanisms of progression of non-alcoholic steatohepatitis. *Trends Mol. Med.* 2008, 14, 72-81.

[3] Tilg, H., The role of cytokines in non-alcoholic fatty liver disease. *Dig. Dis.* 2010, 28, 179-185.

[4] Masarone, M., Federico, A., Abenavoli, L., Loguercio, C. et al., Non alcoholic fatty liver: epidemiology and natural history. *Rev. Recent Clin.Trials* 2014, 9, 126-133.

[5] Arrese, M., Cabrera, D., Kalergis, A. M. & Feldstein, A. E., Innate immunity and inflammation in NAFLD/NASH. *Dig. Dis. Sci.* 2016, 61, 1294-1303.

[6] Tosello-Trampont, A.-C., Landes, S. G., Nguyen, V., Novobrantseva, T. I. et al., Kuppfer cells trigger nonalcoholic steatohepatitis development in diet-induced mouse model through tumor necrosis factor-α production. *J. Biol. Chem.* 2012, 287, 40161-40172.

[7] Rolla, S., Alchera, E., Imarisio, C., Bardina, V. et al., The balance between IL-17 and IL-22 produced by liver-infiltrating T-helper cells critically controls NASH development in mice. *Clin. Sci.* 2016, 130, 193-203.

[8] Guebre‐Xabier, M., Yang, S., Lin, H. Z., Schwenk, R. et al., Altered hepatic lymphocyte subpopulations in obesity‐related murine fatty livers: potential mechanism for sensitization to liver damage. *Hepatology* 2000, 31, 633-640.

[9] Ma, C., Kesarwala, A. H., Eggert, T., Medina-Echeverz, J. et al., NAFLD causes selective CD4+ T lymphocyte loss and promotes hepatocarcinogenesis. *Nature* 2016, 531, 253-257.

[10] Simon, V., Cota, D., Mechanisms in endocrinology: endocannabinoids and metabolism: past, present and future. *Eur. J. Endocrinol.* 2017, 176, R309-R324.

[11] Osei-Hyiaman, D., Liu, J., Zhou, L., Godlewski, G. et al., Hepatic CB 1 receptor is required for development of diet-induced steatosis, dyslipidemia, and insulin and leptin resistance in mice. *J. Clin. Investi.* 2008, 118, 3160-3169.

[12] Irungbam, K., Churin, Y., Matono, T. et al., Cannabinoid receptor 1 knockout alleviates hepatic steatosis by downregulating perilipin 2. *Lab. Invest.* 2020, **1**00, 454-465.

[13] Jorgačević, B., Vučević, D., Samardžić, J. et al., The Effect of CB1 Antagonism on Hepatic Oxidative/Nitrosative Stress and Inflammation in Nonalcoholic Fatty Liver Disease. *Curr. Med. Chem.* 2020 doi: 10.2174/092987327666200303122734. Online ahead of print.

[14] Müller, G., Wied, S., Herling, A., Analysis of Direct Effects of the CB1 Receptor Antagonist Rimonabant on Fatty Acid Oxidation and Glycogenolysis in Liver and Muscle Cells in vitro. *Biochemistry (Moscow)* 2019, 84, 954-962.

[15] Chen, C. C., Lee, T. Y., Kwok, C. F. et al., Major urinary protein 1 interacts with cannabinoid receptor type 1 in fatty acid-induced hepatic insulin resistance in a mouse hepatocyte model. *Biochem. Biophys. Res. Commun.* 2015, 460, 1063-1068.

[16] Chen, C. C., Lee, T. Y., Kwok, C. F. et al., Cannabinoid receptor type 1 mediates high-fat diet-induced insulin resistance by increasing forkhead box O1 activity in a mouse model of obesity. *Int. J. Mol. Med.* 2016, 37, 743-754.

[17] Gary‐Bobo, M., Elachouri, G., Gallas, J. F., Janiak, P. et al., Rimonabant reduces obesity‐associated hepatic steatosis and features of metabolic syndrome in obese Zucker fa/fa rats. *Hepatology.* 2007, 46, 122-129.

[18] Galiègue, S., Mary, S., Marchand, J., Dussossoy, D. et al., Expression of central and peripheral cannabinoid receptors in human immune tissues and leukocyte subpopulations. *Eur. J. Biochem.* 1995, 232, 54-61.

[19] Duncan, M., Galic, M. A., Wang, A., Chambers, A. P. et al., Cannabinoid 1 receptors are critical for the innate immune response to TLR4 stimulation. *Am. J. Physiol. Regul. Integr. Comp. Physiol.* 2013, 305, R224-R231.

[20] Kleiner, D. E., Brunt, E. M., Van Natta, M. et al., Design and validation of a histological scoring system for nonalcoholic fatty liver disease. *Hepatology* 2005, 41, 1313-1321.

[21] Chen, C. C., Lee, T. Y., Kwok, C. F. et al., Using proteomics to discover novel biomarkers for fatty liver development and response to CB1R antagonist treatment in an obese mouse model. *Proteomics* 2017, 17, 1600292.

[22] Arthur, J. S. C., Ley, S. C., Mitogen-activated protein kinases in innate immunity. *Nat. Rev. Immunol.* 2013, 13, 679-692.

[23] McGettigan, B., McMahan, R., Orlicky, D., Burchill, M. et al., Dietary lipids differentially shape nonalcoholic steatohepatitis progression and the transcriptome of Kupffer cells and infiltrating macrophages. *Hepatology* 2019, 70, 67-83.

[24] Day, C. P., James, O. F., Steatohepatitis: a tale of two “hits”? Gastroenterology 1998, 114, 842-845.

[25] Mai, P., Yang, L., Tian, L., Wang, L. et al., Endocannabinoid system contributes to liver injury and inflammation by activation of bone marrow–derived monocytes/macrophages in a CB1-dependent manner. *J. Immunol.* 2015, 195, 3390-3401.

[26] Carbone, F., La Rocca, C., Matarese, G., Immunological functions of leptin and adiponectin. *Biochimie* 2012, 94, 2082-2088.

[27] Hui, J. M., Hodge, A., Farrell, G. C., Kench, J. G. et al., Beyond insulin resistance in NASH: TNF‐α or adiponectin? *Hepatology* 2004*,* 40, 46-54.

[28] Ge, Q., Maury, E., Rycken, L., Gérard, J. et al., Endocannabinoids regulate adipokine production and the immune balance of omental adipose tissue in human obesity. *Int. J. Obes.* 2013*,* 37, 874-880.

[29] Sanches, S. C. L., Ramalho, L. N. Z., Augusto, M. J., da Silva, D. M. et al., Nonalcoholic steatohepatitis: a search for factual animal models. *Biomed. Res. Int.* 2015, 2015, 574832.

[30] Elinav, E., Pappo, O., Sklair‐Levy, M., Margalit, M. et al., Amelioration of non‐alcoholic steatohepatitis and glucose intolerance in ob/ob mice by oral immune regulation towards liver‐extracted proteins is associated with elevated intrahepatic NKT lymphocytes and serum IL‐10 levels. *J. Pathol.* 2006*,* 208, 74-81.

[31] Ni, Y., Nagashimada, M., Zhuge, F., Zhan, L. et al., Astaxanthin prevents and reverses diet-induced insulin resistance and steatohepatitis in mice: A comparison with vitamin E. *Sci. Rep.* 2015, 5, 17192.

[32] Onodera, T., Fukuhara, A., Shin, J., Hayakawa, T. et al., Eicosapentaenoic acid and 5-HEPE enhance macrophage-mediated Treg induction in mice. *Sci. Rep.* 2017, 7, 1-11.

[33] Rajesh, M., Bátkai, S., Kechrid, M., Mukhopadhyay, P. et al., Cannabinoid 1 receptor promotes cardiac dysfunction, oxidative stress, inflammation, and fibrosis in diabetic cardiomyopathy. *Diabetes* 2012, 61, 716-727.

[34] Ryberg, E., Larsson, N., Sjögren, S. et al., The orphan receptor GPR55 is a novel cannabinoid receptor. *Br. J. Pharmacol.* 2007, 152, 1092-1101.

[35] Kapur, A., Zhao, P., Sharir, H. et al., Atypical responsiveness of the orphan receptor GPR55 to cannabinoid ligands. *J. Biol. Chem.* 2009, 284, 29817-29827.

[36] Fondevila, M. F., Fernandez, U., Gonzalez‐Rellan, M. J. et al.,The L‐α‐lysophosphatidylinositol/GPR55 system induces the development of non‐alcoholic steatosis and steatohepatitis. *Hepatology* 2020 doi: 10.1002/hep.31290. Online ahead of print.

[37] Christensen, R., Kristensen, P. K., Bartels, E. M. et al., Efficacy and safety of the weight-loss drug rimonabant: a meta-analysis of randomised trials. *Lancet* 2007, 370, 1706-1713.

[38] Rodgers, R., Evans, P. & Murphy, A., Anxiogenic profile of AM-251, a selective cannabinoid CB1 receptor antagonist, in plus-maze-naive and plus-maze-experienced mice. *Behav. Pharmacol.* 2005, 16, 405-413.

[39] Sink, K. S., Segovia, K. N., Sink, J. et al., Potential anxiogenic effects of cannabinoid CB1 receptor antagonists/inverse agonists in rats: comparisons between AM4113, AM251, and the benzodiazepine inverse agonist FG-7142. *Eur. Neuropsychopharm.* 2010, 20, 112-122.

**Figure Legends**

**Figure 1.** Effects of AM251 on tissue morphology and histopathological changes in the liver tissue of *db/db* mice. Liver tissues from the Lean control mice and *db/db* mice treated with or without 5 mg/kg AM251 were fixed in 10 % formalin for H&E and IHC stain against CD11b (macrophages) and Neutrophils. Results for the H&E stain and IHC stain are shown at 100 x and 200 x magnification, and the dark brown color indicates the sites of CD11b and Neutrophils, respectively. The pathological lesion score of NAFLD was performed according to histological scoring system for NAFLD [20] and the images of CD11b and Neutrophils IHC stains at 200 x magnification were quantified using Panthera L Smart Light Microscope system. ^*^ p < 0.05 vs. Lean control mice (n = 8); ^†^ p < 0.05 vs. *db/db* mice (n = 8).

**Figure 2.** Effects of AM251 on the hepatic levels of CB1 in *db/db* mice. (A) Representative result of the CB1 IHC stain in liver tissues of the Lean control mice and *db/db* mice treated with or without 5 mg/kg AM251. Dark brown color indicates the site of CB1, and the results are shown at 200 x magnification. The score of images was quantified using Panthera L Smart Light Microscope system. (B) The mRNA and (C) protein levels in the liver tissues of mice were detected using quantitative real-time PCR and western blot, respectively. ^*^ p < 0.05 vs. Lean control mice (n = 8); ^†^ p < 0.05 vs. d*b/db* mice (n = 8).

**Figure 3.** Effects of AM251 on lipid droplet accumulation and lipogenesis-regulated factors in the liver tissues of the *db/db* mice. (A) Liver tissues were embedded in an OCT medium and stained with Oil-Red O reagent. The conditions were listed as a: Lean control mice, b: *db/db* mice, c: *db/db* mice treated with 5 mg/kg AM251. The results are shown at 200 x magnification. (B) Liver TG levels were detected using colorimetric assay. (C) The mRNA levels of lipogenesis-regulated factors were measured using quantitative real-time PCR. ^*^ p < 0.05 vs. Lean control mice (n = 7); ^†^ p < 0.05 vs. *db/db* mice (n = 8). TG, triglycerides; SREBP-1, sterol regulatory element-binding factor-1; ACC-1, acetyl-CoA carboxylase-1; FAS, fatty acid synthase; SCD-1, stearoyl-CoA desaturase-1.

**Figure 4**. Effects of AM251 on the inflammatory response and mitogen-activated protein kinase (MAPK)-related factor levels in the liver tissues of *db/db* mice. (A) Hepatic mRNA levels of TNF-α, IL-6, MCP-1, and IFNγ were measured by quantitative real-time PCR. (B) Hepatic protein phosphorylation levels of p-38, ERK, and JNK were detected using western blot. ^*^ p < 0.05 vs. Lean control mice (n = 7); ^†^ p < 0.05 vs. *db/db* mice (n = 8). TNF-α, tumor necrosis factor-α; IL-6, interleukin-6; MCP-1, monocyte chemoattractant protein-1; IFNγ, interferon γ; ERK, extracellular signal-regulated kinase; JNK, c-Jun N-terminal kinase.

**Figure 5.** Flow cytometry analysis of the macrophages, T helper cells, natural killer T (NKT) cells, and regulatory T (Treg) cells in the Lean control mice and *db/db* mice treated with or without 5 mg/kg AM251. HMNCs were isolated from fresh liver tissues of mice prior to the execution of flow cytometry analysis. The axes on the flow cytometry dot-plots were labeled at Trapezoid shape with black color or round shape with red color. The expression of F4/80, CD3^+^CD4^+^, CD3^+^NK1.1^+^, CD4^+^CD25^+^ was presented as a percentage of macrophages, T helper cells, NKT cells and Treg cells, respectively. ^*^ p < 0.05 vs. Lean control mice (n = 8); ^†^ p < 0.05 vs. *db/db* mice (n = 8).

**Figure 6.** Effects of AM251 on HFFA or ACEA-induced CB1 protein levels and the inflammatory response in RAW264.7 cells. (A and C) The CB1 protein expression and (B and D) cellular mRNA levels of TNF-α, IL-6, MCP-1, and IFNγ were analyzed using western blot and quantitative real-time PCR, respectively. Raw264.7 cells were pretreated with 3.3 μM AM251 for 1 h, followed by co-treatment of 3.3 μM AM251 with 1 mM HFFA or 1 μM ACEA for 24 h, separately. Data are presented as the mean ± S.E.M. from three independent experiments. ^*^ p < 0.05 vs. HFFA (-); ^†^ p < 0.05 vs. HFFA (+); ^§^ p < 0.05 vs. ACEA (-); ^¥^ p < 0.05 vs. ACEA (+). TNF-α, tumor necrosis factor-α; IL-6, interleukin-6; MCP-1, monocyte chemoattractant protein-1; IFNγ, interferon γ.

**Figure 7**. Effects of genetic silencing of CB1 on HFFA- or ACEA-induced inflammatory responses in the RAW264.7 cells. (A) Changes in expression of the CB1 protein after CB1 siRNA transfection in RAW264.7 cells. (B and C) Cellular mRNA levels of TNF-α, IL-6, MCP-1, and IFNγ were measured after CB1 gene silencing, followed by exposure to 1 mM HFFA or 1 μM ACEA for 24 h. Values are presented as the mean ± S.E.M. from three independent experiments. ^*^ p < 0.05 vs. HFFA (-); ^†^ p < 0.05 vs. HFFA (+); ^§^ p < 0.05 vs. ACEA (-); ^¥^ p < 0.05 vs. ACEA (+). TNF-α, tumor necrosis factor-α; IL-6, interleukin-6; MCP-1, monocyte chemoattractant protein-1; IFNγ, interferon γ.

**Supplementary Figure 1.** Comparison of (A) body weight and (B) food intake in the Lean control mice and *db/db* mice treated with or without 5 mg/kg AM251. Fifteen-week-old mice fed with a standard diet and water *ad libitum.* Day 0 was defined in prior to the AM251 or vehicle solution treatment, then mice were administrated with AM251 or vehicle solution for 15 days. The body weight and food intake of mice were measured for every 3 days and every day, respectively. * p < 0.05 vs. Lean control mice (n = 14); † p < 0.05 vs. *db/db* mice (n = 14).
